# Study on antihepatocellular carcinoma effect of 6-shogaol and curcumin through network-based pharmacological and cellular assay

**DOI:** 10.3389/fphar.2024.1367417

**Published:** 2024-08-19

**Authors:** Qiuxia Jin, Wenya Jiao, Yunhe Lian, Bimal Chitrakar, Yaxin Sang, Xianghong Wang

**Affiliations:** ^1^ College of Food Science and Technology, Hebei Agricultural University, Baoding, China; ^2^ Chenguang Biotechnology Group Co., Ltd., Handan, China

**Keywords:** 6-shogaol, curcumin, hepatocellular carcinoma, synergistic effect, PI3/AKT signaling pathway, MAPK signaling pathway

## Abstract

**Background:**

Hepatocellular carcinoma currently has the third highest mortality rate in the world. Patients with hepatocellular carcinoma are on the rise and at a younger age, but research into the pharmacological effects of cancer is mostly single-component, and natural plant products can have additive or synergistic effects that can better amplify the effects of intervention in cancer.

**Aim:**

To evaluate the synergistic therapeutic effects of 6-shogaol and curcumin against hepatocellular carcinoma line HepG2 cells.

**Methods:**

In this study, a network pharmacology approach was used to predict and validate the mol ecular targets and pathways of the hepatocellular carcinoma (HCC) of 6-shogaol and curcumin in combination and to investigate their mechanism of action. The results were also validated by cellular assays. HepG2 cells were treated with 6-shogaol and curcumin as well as the combination of the two. The combination index of 6-shogaol and curcumin in HepG2 cells was calculated using Compusyn software according to the Chou-Talalay equation. The synergistic anti-cancer effect was next investigated by MTT assay, apoptosis assay and cell cycle assay. The combined anti-hepatocellular carcinoma effect of the Ras-mediated PI3K/AKT and MAPK signalling pathways was analysed using protein blotting assays.

**Results:**

A network pharmacology-based screening identified 72 core targets of 6-curcumin and curcumin in hepatocellular carcinoma, and predicted that the main signalling pathway is the Ras signalling pathway. The anti-cancer effects of 6-shogaol and curcumin were validated in cell-based assays and the optimal synergistic concentrations of 5 μmoL/L for 6-shogaol and 30 μmoL/L for curcumin were determined. 6-shogaol and curcumin synergistically blocked the cell cycle in the G2/M phase and promoted apoptosis. Immunoblot analysis confirmed for the first time the combined action of both in down-regulating the Ras-mediated PI3K/AKT and MAPK signaling pathways. In addition, 6-shogaol and curcumin acting together downregulated Cyclin-B, CDK-1, Bcl-2, and upregulated BAX.

**Conclusion:**

6-shogaol and curcumin act synergistically to alter the morphology of hepatocellular carcinoma cells, block the cell cycle in the G2/M phase, inhibit proliferation and division, and effectively promote late apoptosis. The combined action of these two components provides a theoretical basis for the further development of novel anti-liver cancer products.

## 1 Introduction

Hepatocellular carcinoma (HCC) is one of the common malignancies; it is the fifth highest incidence of malignancies with the second highest mortality rate (Couri and Pillai, 2019). According to the data released by the National Cancer Center of China, in 2022, the number of primary liver cancer cases nationwide will be 367,700, ranking fourth in the number of new cases of various types of cancer (lung, colorectal, thyroid, and liver), and the incidence rate will be fifth (lung, female breast, thyroid, colorectal, and liver); and in 2022, the number of deaths due to primary liver cancer will be 316,500, ranking second in terms of both the number of deaths and the mortality rate (lung, liver) (Zheng et al., 2022). To date, chemotherapy is the common treatment but it is not as effective as it could be due to the adverse side effects as well as drug resistance (Ikeda et al., 2018). Medicinal and food-based substances usually exert chemo-preventive effects on diseases in a non-toxic form. In recent years, natural product-related drugs have accounted for a large proportion of the current clinically used anticancer drugs; the development of novel antitumour drugs has received increasing attention. The combined use of different plant extracts can have significant anticancer effects or physiological effects (Wang et al., 2015; Liu et al., 2022).

**FIGURE 1:**
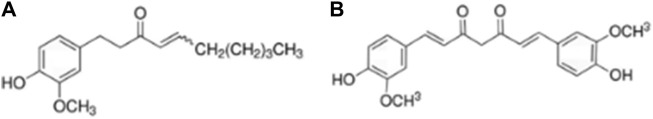
Chemical structural formula of 6-shogaol and curcumin. **(A)** 6-shogaol. **(B)** curcumin.

Dried ginger is the rhizome of ginger family, which is commonly used worldwide as a spice and herb. The chemical composition of ginger is complex, containing 194 volatile oils (mostly aliphatic hydrocarbons), 85 curcuminoids and 28 diphenyl-heptanes (Dalsasso et al., 2022; Ivane et al., 2022). Studies on the active components and functions of ginger have focused on ginger enol, and ginger essential oil, with less research on ginger proteins and ginger polysaccharides (Liu et al., 2019). 6-Shogaol (Figure 1A), a type of gingerol, is derived from the dehydration of 6-gingerol and is found in low levels in ginger but high levels in dried and canned ginger products. Curcumin (Figure 1B) is a fat-soluble polyphenol consisting mainly of two benzene rings substituted by hydroxyl and methoxy groups, linked by a keto-enol structure with seven carbon atoms.

6-shogaol is the main active constituent of ginger and curcumin is an important active ingredient in ginger which have various pharmacological effects such as antitumor, anti-inflammatory, antioxidant, hypoglycemic and hypolipidemic (Zhou et al., 2022). Numerous studies have been conducted on the anti-tumor activities of both 6-shogaol and curcumin; for example, 6-shogaol can significantly inhibit the progression of a variety of malignant tumours (Lung and breast cancer) by reducing the release of CC-chemokine ligand (CCL-2) from tumour-associated dendritic cells (Hsu et al., 2015); 6-shogaol inhibits cell autophagy by inducing G2/M phase cell cycle block and by targeting Notch signalling pathway to inhibit cellular autophagy exacerbating apoptosis, thereby inhibiting breast cancer progression (Bawadood et al., 2020); it can also inhibit apoptosis in hepatocellular carcinoma cells by targeting p53, thereby effectively enhancing the sensitivity of hepatocellular carcinoma cells to TRAIL-induced apoptosis (Nazim and Park, 2019). Additionally, curcumin can inhibit tumor interstitial cell death by inhibiting the IL-6/ERK/NF-κB pathway to inhibit tumor mesenchymal crosstalk and pancreatic cancer metastasis and also inhibit HCC as well as improve drug resistance (Li et al., 2020); curcumin inhibits human hepatocellular carcinoma cell invasion and metastasis through Bclaf1-mediated Wnt/β-catenin signalling (Zhao et al., 2022); curcumin inhibits PI3K/AKT/GSK-3β pathway activation and triggered apoptosis in HCC mitochondria (Bai et al., 2022). Many molecular pathways have been implicated in HCC carcinogenesis, including TERT promoter mutations, Wnt/β-linked protein, P53, Akt/mTOR, vascular endothelial growth factor receptor (VEGFR) and endothelial growth factor receptor (EGFR)/RAS/MAPK pathways (Zhang et al., 2020). This study therefore aims to further define the anti-HCC effect of the combination of 6-shogaol and curcumin and to elucidate its mechanism of action.

Network pharmacology is an interdisciplinary assay that uses system biology to explain the relationship between drug components, targets, and diseases (Tang et al., 2022). Network pharmacology emphasizes the multi-pathway regulation of signaling pathways to reduce toxic side effects and improve the therapeutic effects of drugs, thus increasing the success rate of clinical trials of new drugs and saving the cost of drug research and development.2021 On 9 March 2021, the World Federation of Societies of Traditional Chinese Medicine (WFSTCM) certified and adopted the “Guidelines on Evaluation Methods in Network Pharmacology”. The development of this guideline is conducive to the improvement of the overall research level in the field of network pharmacology research, and has important practical guidance significance for standardizing the research of network pharmacology discipline. In Chinese medicine, network pharmacology emphasizes the analysis of molecular association between drugs and therapeutic objects from the perspective of system level and biological network as a whole, and its research concept coincides with the holistic thinking of Chinese medicine, which has been widely used in the discovery of active compounds of drugs and traditional Chinese medicines, the interpretation of the overall mechanism of action, and the analysis of the rules for the combination of drugs and prescriptions, providing new ideas for the study of the complex system of traditional Chinese medicines, and providing new guidance for the rational use of clinical drugs. It has provided new ideas for the research of complex system of Chinese medicine, and provided new scientific and technological support for the rational use of medicine in clinic and the research and development of new drugs (Hu and Sun, 2017). There are four main types of databases in network pharmacology: 1) Chinese medicine databases, mainly for obtaining the ingredients of Chinese medicines, some of which contain their targets. For example, TCMSP, ETCM, HERB, BATMAN-TCM, TCMID, SymMap, TCM-ID, and anti-tumor natural product database. 2) Databases for obtaining compound information, collecting various information of Chinese medicine compounds. For example, Pubchem, Swiss ADME, Chinese Academy of Sciences Compound Reference Database, ProTox-II - Prediction of TOXicity of chemicals, DrugCentral, STITCH and so on. 3) Disease database summarization, mainly used to collect disease-related targets. For example: GeneCards, OMIM, Drugbank, TTD, DisGeNET, Malacards, etc. Chou-Talalay (Median pharmacodynamic method; a combination index method) is a quantitative method for drug combination, established by Chou and Talalay in 1984; it has the advantages of scientific principles with complete mathematical models. It is widely used in antitumor drug combination studies for its scientific principles, well-developed mathematical models and simple experimental operation (Kifer et al., 2020). The main objective of this study was to investigate the synergistic effects and to compare the antihepatocellular carcinoma effects of 6-shogaol and curcumin individually or in combination.

Dried ginger and its components for the treatment of HCC have been shown to inhibit the proliferation of HCC cells but the combination of monomeric components has not been widely reported. Therefore, we chose a combination of two monomers, namely 6-shogaol and curcumin in this study with an aim that a lower concentration could be used if the two compounds had synergistic effects, thus providing a new experimental and theoretical basis for clinical treatment. The HepG2 cell line was then subjected to *in vitro* experiments to identify the identified molecular targets and metabolic mechanisms. The results of this study are expected to facilitate the integrated use and proliferation of 6-shogaol and curcumin in functional food, pharmaceutical and nutraceutical industries.

In addition to the preliminary results indicating possible synergies, the expected interactions between 6-shogaol and curcumin in cell cycle, apoptosis and proliferation make this combination particularly worthy of further investigation. It has promising applications in the development of new drugs (Plant extracts and active ingredients) and in conducting medicinal studies (Zhou et al., 2016).

## 2 Materials and methods

### 2.1 Materials and chemical reagents

MTT cell proliferation and cytotoxicity assay kits were purchased from SolarBio Technology Ltd. (Beijing, China). Cell cycle kits and apoptosis kits were purchased from Hangzhou Unitech Biotechnology Co. (Hangzhou, China). Antibodies, namely PI3K, AKT, Ras, MEK_1/2_, ERK_1/2_, Cyclin-B, CDK-1, Caspase-8, Bax, and Bcl-2 were purchased from Abcam (Cambridge, UK). Human hepatocellular carcinoma HepG2 cells (Procell CL-0103) were kindly provided by Procell Life Science and Technology Co. (Wuhan, China). 6-shogaol (Analytical standard, HPLC≥98%) and curcumin (Analytical standard, HPLC≥98%) specimens were purchased from Shanghai Yuanye Biotechnology Co., Ltd. (Shanghai, China).

### 2.2 Network pharmacology

#### 2.2.1 Prediction of the target of action of 6-shogaol and curcumin

Search for potential targets of action of 6-shogaol and curcumin in humans from internet databases, including TCMSP (http://tcmspnw.com/), SwissTargetForecast (http://swisstargetprediction.ch/), and Pharmmapper (http://www.lilab-ecust.cn/pharmmapper/). The targets from the above sources were combined and used as relevant component targets for 6-shogaol and curcumin after removing redundancy.

#### 2.2.2 Prediction of targets relevant to hepatocellular carcinoma

The search for disease targets related to hepatocellular carcinoma was conducted from DisGenet (https://www.disg enet. org/), Genecards (https://www.genecards.org/), OMIM (https://omim.org/), TTD (http://ttd.org/) and DrugBank (http://ttd.org/ drugbank. com/) web databases using the keyword “Hepatocellular carcinoma”. After combining these targets, duplicate genes were eliminated and used as targets for hepatocellular carcinoma-related diseases.

The UniProt database (https://www.uniprot.org/) was used to screen the component targets and disease targets, limited to the species “*Homo sapiens*” and “Reviewed”; these were normalised to target genes. Constituent targets and disease targets were imported into the Draw Venn website (http://bioinformatics.psb.ugent.be/webtools/Venn/) to obtain relevant targets for constituent-acting diseases.

#### 2.2.3 Protein-protein interaction network diagram

The STRING (https://string-db.org) database was used to obtain PPI data for 6-shogaol and curcumin targets in hepatocellular carcinoma, where the parameter genus was set to “*Homo Sapiens*”; the confidence level was set to high confidence “0.7"; and other parameters were set to default values. Cytoscape 3.7.2 software was applied to build PPI relationship networks and perform topological analysis to build component-target interaction network and protein-protein (PPI) interaction network maps for the effects of 6-shogaol and curcumin on hepatocellular carcinoma.

#### 2.2.4 GO enrichment and KEGG enrichment

The KEGG pathway and GO biological processes were enriched using the DAVID 2021 database (https://david.ncifcrf.gov/). Signalling pathway analysis was performed using the KEGG database. The pathways with *p*-values≤0.05 and Benjamin values ≤ 0.05 were selected for functional annotation clustering, resulting in the top ranked biological processes and 15 pathways of interest.

The GO enrichment analysis included cellular component, molecular function and biological process. Gene function was interpreted from these three aspects and entries with *p* < 0.05 were collected for annotation clustering. The bioinformatics platform (http://www. bioInformations.com.cn/) was used for visual analysis to produce bubble plots and histograms.

#### 2.2.5 “Target-pathway” network construction

A “target-pathway” network is a network diagram formed by linking a bioactive component to a putative target and signalling pathway. Network maps were formed using predicted targets associated with hepatocellular carcinoma and KEGG signalling pathways. The network was constructed using Cytoscape 3.7.2 software to map the “target-pathway” network.

### 2.3 Cell tests

#### 2.3.1 Cell culture

HepG2 cells were inoculated into T25 bottles and cultured in MEM medium containing 10% FBS (5 mL culture medium per bottle) at 37 °C with 5% CO_2_ and 95% air. The culture medium was changed every other day; when the cells were spread out, they were digested with trypsin for passaged culture (Zhu et al., 2018).

#### 2.3.2 Measurement of cell proliferation inhibition rate

Log phase cells were digested with trypsin and the concentration was adjusted to 5*10^3^ cells/well and inoculated in 96-well plates and cultured in MEM containing 10% FBS (100 μL of culture medium per well). After 24 h of cell apposition, the cells were replaced with MEM containing 10% fetal bovine serum at different concentrations of 6-shogaol and curcumin to treat the cells individually. After 24, 48 and 72 h of incubation, 20 μL of MTT solution was added to each well; after 4 h of incubation, the supernatant was aspirated off and 150 μL of Formazan lysate was added to each well and placed on a shaker for 10 min at low speed to fully dissolve the crystals. The absorbance (OD) of each well was measured at 570 nm by Microplate reader (Sani et al., 2015). Each experiment was repeated three times. The effect of each component on the viability of HepG2 cells and the half-inhibitory concentration (IC_50_) were calculated. Cell viability was calculated according to the following formula Equation 1:

$$\text{Cell survival rate }\left(\text{Fu}\right)=\frac{\left(\text{OD}\;\left(\text{experimental}\;\text{group}\right)\;-\;\text{OD}\;\left(\text{blank}\;\text{group}\right)\right.}{\text{OD}\;\left(\text{control}\;\text{group}\right)\;-\;\text{OD}\;\left(\text{blank}\;\text{group}\right)}*100\%$$ (1)

#### 2.3.3 Combination index analysis index analysis

The Chou-Talalay combination index method was used to analyse the interaction of the two components, either as a single component or as a mixture of the two components in a fixed ratio. Drug synergy studies were performed using Compusyn software version 1.0 (Ting Chao Chou and Nick Martin, Paramus, NJ, 2005), where the combination index (CI) was measured and the dose effect curve, combination index and dose reduction index (DRI) were plotted. The CI was calculated and the interaction of the two components was determined according to the following formula.

CI = $\frac{D_{1}}{D_{x1}}$ + $\frac{D_{2}}{D_{x2}}$ , where, $D_{x1}$ and $D_{x2}$ represent the dose required to achieve the same efficacy for the combination of component 1 and component 2 as for component 1 ( $D_{1}$ ) and component 2 ( $D_{2}$ ) alone (Dewangan et al., 2017).

#### 2.3.4 Cell apoptosis assay

HepG2 cells at logarithmic growth stage were inoculated in 6-well plates at 1 × 10^5^ cells/well. After cell apposition for 24 h, cells were replaced with MEM containing 10% fetal bovine serum and 5 μM 6-shogaol and 30 μM curcumin alone and in combination for 24 h. The cells were observed for apoptotic or dead morphology, observed under a Nikon light microscope and photographed. After digestion with EDTA-free trypsin, the cells were washed 3 times by centrifugation with pre-cooled PBS and the supernatant was discarded. 500 μL 1x Binding buffer was taken to resuspend the cells. Then, 5 μL AnnexinV-FITC and 10 μL PI were added and mixed gently; after incubation for 5 min at room temperature without light, the apoptosis rate was detected by flow cytometry (Kumar et al.,2018).

#### 2.3.5 Cell cycle assay

HepG2 cells at logarithmic growth stage were inoculated in 6-well plates at 1 × 10^5^ cells/well. After cell walling for 24 h, the cells were treated with MEM containing 10% fetal bovine serum and 5 μM 6-shogaol and 30 μM curcumin alone and in combination for 24 h. After digestion with EDTA-free trypsin, the cells were collected; fixed in ethanol; washed with PBS overnight at 4°C; and then treated with RNA. The RNA was removed with RNase A; fluorescently labelled; and then assayed on the machine (Specian et al., 2016).

#### 2.3.6 Western blot assay

HepG2 cells at logarithmic growth stage were inoculated in 6-well plates at 1 × 10^5^ cells/well. After 24 h of cell walling for 24 h, the cells were treated with MEM containing 10% fetal bovine serum and 5 μM 6-shogaol and 30 μM curcumin alone and in combination for 24 h. The original culture medium was poured off; washed with pre-cooled PBS; and blotted up. Then, phosphatase inhibitor cocktail (100×) and RIPA lysis buffer (Stron) were added at a ratio of 1:99. The cells were incubated on ice for 20 min to allow sufficient lysis; the mixture was centrifuged; and the supernatant was removed. The protein concentration in the supernatant was measured using the BSA kit. The protein solution was then mixed with 5*SDS-PAGE loaded protein with buffer in a 4:1 ratio and boiled for 5 min. The target proteins were transferred to NC membranes by gel electrophoresis and detected by ultrasensitive chemiluminescent chromogenic solution after the addition of anti-monoclonal antibodies and corresponding secondary antibodies (Jiao et al., 2021). Protein signals were visualised and quantified using ImageJ software (National Institute of Health, USA; version 1.8.0_112).

### 2.4 Data processing and statistical analysis

The data obtained were analysed using Graphpad Prism 6 and data were expressed as mean ± standard deviation. One-way ANOVA was used for comparisons between multiple groups and t-tests were used for two-way comparisons between groups. Differences at the 95% confidence level (*p* < 0.05) were considered statistically significant.

## 3 Results

### 3.1 Network pharmacological predictions

#### 3.1.1 6-shogaol and curcumin-related target prediction

The targets related to 6-shogaol and curcumin were screened by TCMSP, SwissTargetForecast and Pharmmapper databases. The numbers of targets associated with 6-shogaol were 7, 100 and 125, respectively, and 208 targets were obtained after eliminating duplicates; the numbers of targets associated with curcumin were 0, 64 and 107, respectively, and 162 targets were obtained after eliminating duplicates. The 6-shogaol and curcumin targets with duplicate sequences removed were combined together, and a total of 272 common targets were obtained after removing duplicate sequences again.

#### 3.1.2 Hepatocellular carcinoma-related target prediction

**FIGURE 2:**
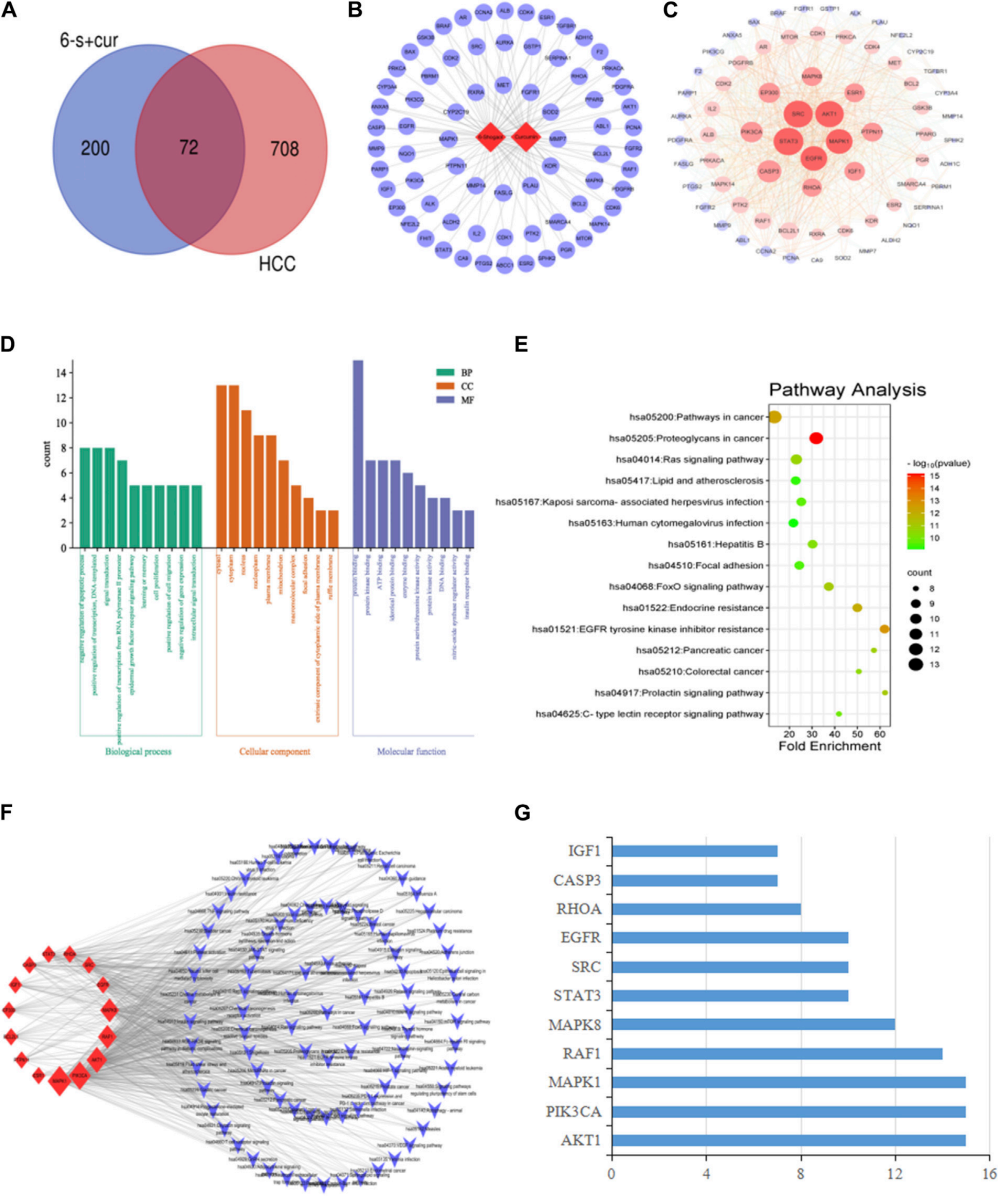
Network pharmacological analysis of 6-shogaol and curcumin for the treatment of hepatocellular carcinoma: **(A)** Venn diagram of component targets *versus* disease targets. **(B)** Constituent-target interactions network diagram. The network depicts the relationship between the component and the target. **(C)** Network diagram of the interaction of 6-shogaol and curcumin with the hepatocellular carcinoma target gene PPI. Correlation is represented by colour and target size. **(D)** GO enrichment analysis. The length and colour of the bands were used to describe the number of targets involved in the biological process of interest. **(E)** Bubble diagram of KEGG pathway analysis. The top 15 pathways with corresponding *p*-values are shown as dot plots. The colour scale indicates the *p*-value and the dot size represents the number of genes per term. **(F)** Construction of a key target-pathway network. The network describes the relationship between targets and pathways. **(G)** The proportion of key targets in the top 10 pathways. The vertical coordinates indicate the relevant targets in the first fifteen paths, and the horizontal coordinates indicate how many paths the relevant targets are shown in.

For the hepatocellular carcinoma-related targets, a search using the keyword “Hepatocellular carcinoma” yielded 154, 497, 193, 54 and 35 disease targets using the median method, respectively using the Digenet, Genecards, OMIM, TTD and DrugBank databases. Among them, 779 targets were obtained after removing duplicates. These targets were intersected with 272 targets screened in section 3.1.1 and a Venn diagram was made (Figure 2A), yielding a total of 72 intersected targets.

#### 3.1.3 Component-target network diagram

Based on the compounds and predicted targets, we constructed a network consisting of components and targets using Cytoscape 3.7.2 (Figure 2B). The network contained a total of 74 nodes; of which, 2 were compound nodes and 72 were target nodes, forming 108 compound-target associations. The network revealed potential relationships between compounds and targets, thus revealing the potential pharmacological mechanisms of the two components for the treatment of hepatocellular carcinoma. With 62 connections for 6-shogaol and 46 connections for curcumin, these findings suggested that one compound affected multiple targets. Of these potential protein targets, 36 were highly correlated with both components (degree = 2); these results suggested that multiple compounds can target a single gene in an interactive manner, supporting the multi-component, multi-target nature of the inhibitory effects of the components in both stem ginger on hepatocellular carcinoma, and that the high degree of protein targets in these networks may explain the underlying therapeutic effects of both components on hepatocellular carcinoma effects.

#### 3.1.4 Protein-protein interaction network map

Protein-protein interactions were performed using the STRING database for the intersection targets between two component targets (6-shogaol and curcumin), and liver cancer disease targets. The PPI network was constructed using Cytoscape 3.7.2 software; Figure 2C shows that the PPI network consisting of 70 nodes and 832 edges. In the PPI network, higher degree values indicate a stronger possibility of a role in the pharmacological process. Based on the topological nature of the network, the 15 nodes with values greater than the average were selected, based on the magnitude of the relevant parameter values (Degree, betweenness centrality and closeness centrality) were visualised and analysed. The larger the node, the higher the degree of association with other proteins (Table 1). We also hypothesized that these 15 key nodes were involved in multiple pathways and might be the core targets for 6-shogaol and curcumin to inhibit hepatocellular carcinoma.

**TABLE 1:** Top 15 core targets.

Target	Degree	Betweenness Centrality	Closeness
STAT3	36	0.10348	0.65094
SRC	35	0.09512	0.63302
AKT1	34	0.11590	0.61607
MAPK1	31	0.06071	0.6
EGFR	31	0.05647	0.60526
CASP3	27	0.06069	0.6
PIK3CA	24	0.02501	0.54330
EP300	23	0.06925	0.56097
ESR1	21	0.02153	0.552
PTPN11	21	0.01361	0.53076
IGF1	21	0.01716	0.53488
MAPK8	21	0.09731	0.57024
RHOA	20	0.02707	0.54330
BCL2L1	18	0.02115	0.53076
PTK2	17	0.00594	0.50735

Notes: Based on the topological nature of the network, the 15 nodes with values greater than the average were selected, based on the magnitude of the relevant parameter values (Degree, betweenness centrality and closeness centrality) were visualised and analysed.

#### 3.1.5 GO enrichment and KEGG enrichment analysis

To explore the possible mechanism of 6-shogaol and curcumin action on hepatocellular carcinoma, we constructed a bubble map as shown in Figure 2D. It contained several signalling pathways related to cancer, such as Ras-, Foxo-, Prolactin-, C-type lectin receptor-signalling pathway, etc., among which, the Ras signalling pathway was the most important. GO enrichment analysis was then performed on 72 common targets using the DAVID 2021 database to elucidate the possible roles of the candidate targets. The results of GO enrichment analysis (Figure 2E) indicated that the key targets for the interaction of 6-shogaol and curcumin with hepatocellular carcinoma were mainly involved in the negative regulation of apoptotic process, positive regulation of transcription, signal transduction, RNA polymerase II promoter transcription positive regulation, epidermal growth factor receptor signaling pathway, learning and memory, cell proliferation, gene expression, and other biological processes. They are involved in a variety of functions, including protein binding, homologous protein binding, ATP, enzymes, and transcription factors. They are also involved in cellular components, including cytoplasm, nucleus, plasma membrane, and nucleoplasm.

#### 3.1.6 Target-pathway network construction

A total of 15 pathways associated with hepatocellular carcinoma were obtained, based on the correlation results of KEGG signalling pathway enrichment, which were analysed by Cytoscape 3.7.2 software to obtain a target-pathway network interplay map (Figure 2F). This network contained a total of 103 nodes (15 intersecting targets and 88 KEGG pathways). Among these potential pathways, the Ras signalling pathway was considered to be the most important pathway with the highest degree (Degree = 10), which played an important role in cell proliferation and apoptosis. Combining drug target prediction, and GO and KEGG enrichment analysis, it was hypothesized that the effects of 6-shogaol and curcumin on hepatocellular carcinoma might be related to the Ras signaling pathway regulating proliferation and apoptosis of hepatocellular carcinoma cells. To further analyse the core targets (as shown in Figure 2G), we ranked the top 15 pathways involved according to the number of targets in them. The results indicated that AKT1, PIK3CA, and MAPK1 were involved in a wide range of pathways and may be core targets of 6-shogaol and curcumin.

### 3.2 Cellular experiments

#### 3.2.1 6-shogaol and curcumin inhibit the proliferation of HepG2 cells

**FIGURE 3:**
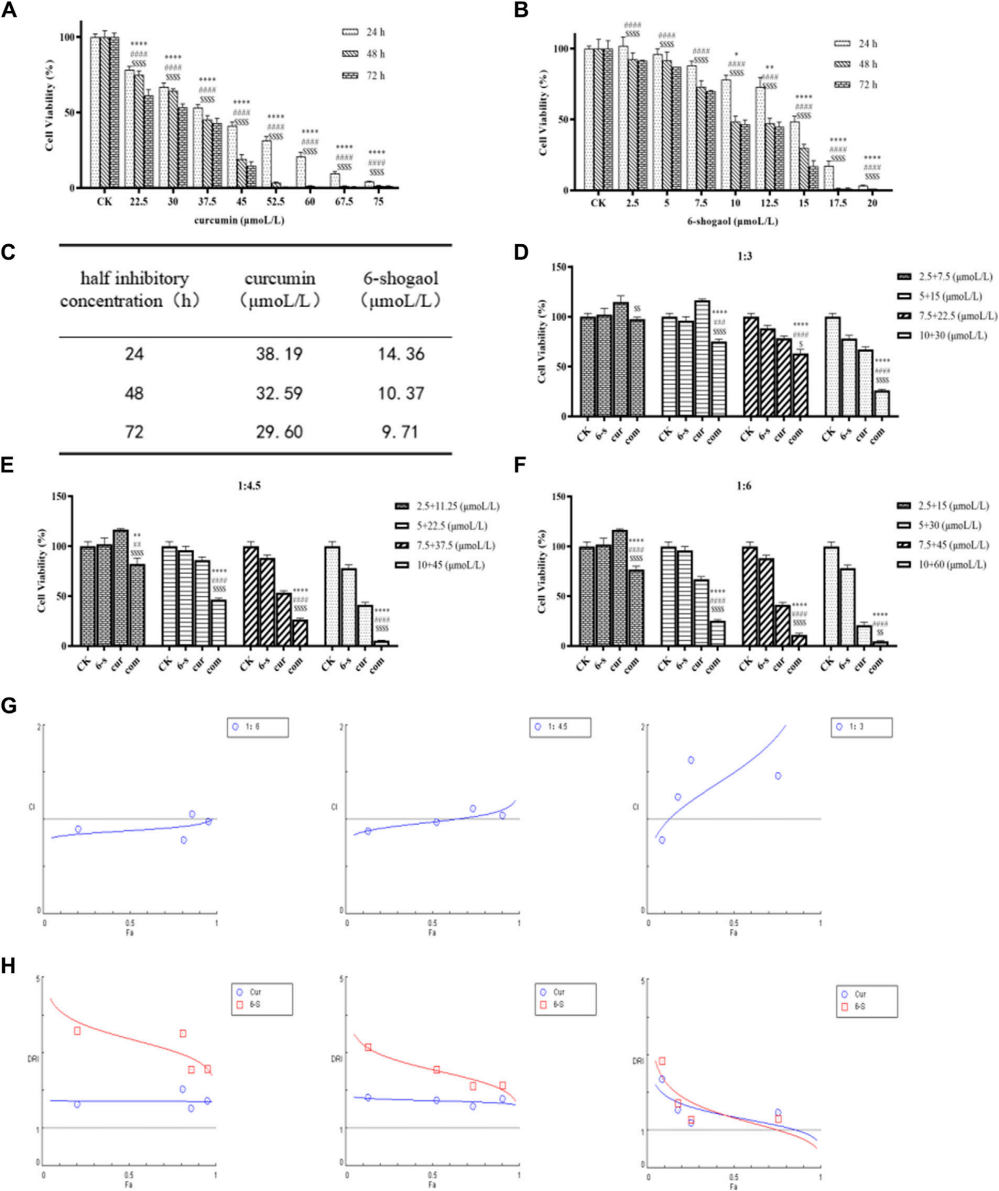
6-shogaol and curcumin inhibited the growth and proliferation of human hepatocellular carcinoma cells. **(A-C)** Effect of 6-Shogaol and curcumin on the proliferation inhibition rate of human hepatocellular carcinoma cells at 24 h, 48 h and 72 h and the change in IC50 values, respectively. Results are expressed as mean ± SD (n = 3). ^*^ *p* < 0.05, ^**^ *p* < 0.01, ^***^ *p* < 0.001, calculated compared with the control group. **(D-F)** Cell viability of different combinations of 6-shogaol and curcumin was assessed by MTT assay. Results are expressed as mean ± SD (n = 3). ^*^ *p* < 0.05, ^**^ *p* < 0.01, ^***^ *p* < 0.001, calculated compared to the control group. **(G)** CI values for 6-shogaol and curcumin were calculated by CompuSyn software at 24 h time points according to the Chou-Talay method. Each circular symbol indicates the CI value of the drug effect (Fa) in HepG2 cells at four different combination ratios. All data are representative of DRI values for the combination of **(H)**6-shogaol and curcumin from three independent experiments. The circles (blue) and rectangles (red) represent the different combination ratios.

To investigate the effect of 6-shogaol and curcumin on the proliferation of HepG2 cells, different doses of 6-shogaol (2.5, 5, 7.5, 10, 12.5, 15, 17.5 and 20 μM) and curcumin (22.5, 30, 37.5, 45, 52.5, 60, 67.5 and 75 μM) alone were used to HepG2 cells, which were treated for 24, 48 and 72 h (Figures 3A,B) and IC_50_ values were calculated (Figure 3C). IC_50_ values can measure the ability of drugs to induce apoptosis; the stronger the induction ability, the lower the value. From Figure 3C, it can be seen that the induction ability of 6-shogaol was greater than that of curcumin; curcumin had cytotoxic effects on HepG2 cells and could inhibit the growth and proliferation of HepG2 cells, and the degree of inhibition was positively correlated with the drug concentration.

#### 3.2.2 Inhibition of HepG2 cell proliferation by the combination of 6-shogaol and curcumin

Drug interaction relationships were studied using Composyn software. When CI < 1; CI = 1; and CI > 1, respectively indicated synergistic, additive and antagonistic effects between drugs, while CI < 0.9 indicated strong synergistic effects. The concentration ratios of 6-shogaol and curcumin were 1:3、1:4.5and 1:6 and it was evident from the results (Figures 3D–F) that the combination of the two drugs was more effective than the single drug effect. A stronger synergistic effect was shown when the concentrations of 6-shogaol and curcumin were 5 μM and 30 μM, compared to the other dose groups, as it produced a lower CI value of 0.77991 (Figure 3G; Table 2). Drug synergy was expressed in terms of combination index and DRI values (Figure 3H; Table 3). The DRI values for each group were greater than 1. This concentration was therefore chosen for the drug administration intervention on HepG2 cells in subsequent experiments.

**TABLE 2:** Combination index values for 6-shogaol and curcumin combinations.

Drug combination (μM 6-shogaol: μM curcumin ratio)	Combination index
2.5 + 7.5 (1:3)	0.79
5 + 15	1.24
7.+22.5	1.62
10 + 30	1.46
2.5 + 11.25 (1:4.5)	0.87
5 + 22.5	0.97
7.5 + 33.75	1.12
10 + 45	1.04
2.5 + 15 (1:6)	0.99
5 + 30	0.78
7.5 + 45	1.05
10 + 60	0.98

Notes: Combination index values were generated by CompuSyn software using formula CI = (D)1/(Dx)1+(D)2/(Dx)2, where (Dx)1 or (Dx)2 represents the dose of drug 1 or 2 in a combination needed for achieving the same efficiency as that of the single drug 1 or 2 at D1 or D2, respectively. CI < 1 indicate drug synergism, CI > 1 antagonism and CI, 1 show additive effect.

**TABLE 3:** Dose reduction indices of drug combinations on 6-shogaol and curcumin in HepG2 cells.

Drug combination (μM 6-shogaol: μM curcumin ratio)	Dose reduction index	
	6-shogaol	Curcumin
2.5 + 7.5 (1:3)	2.8395	2.3491
5 + 15	1.7206	1.5284
7.5 + 22.5	1.2805	1.1848
10 + 30	1.2960	1.4554
2.5 + 11.25 (1:4.5)	3.1520	1.8070
5 + 22.5	2.5404	1.7380
7.5 + 33.7	2.1048	1.5607
10 + 45	2.1248	1.7589
2.5 + 15 (1:6)	3.5825	1.6151
5 + 30	3.5011	2.0231
7.5 + 45	2.5444	1.5180
10 + 60	2.5601	1.7030

Notes: Dose reduction index were generated from CompuSyn software. More than 1 DRI, value show favourable drug combination.

#### 3.2.3 Morphological observation by light microscopy

**FIGURE 4:**
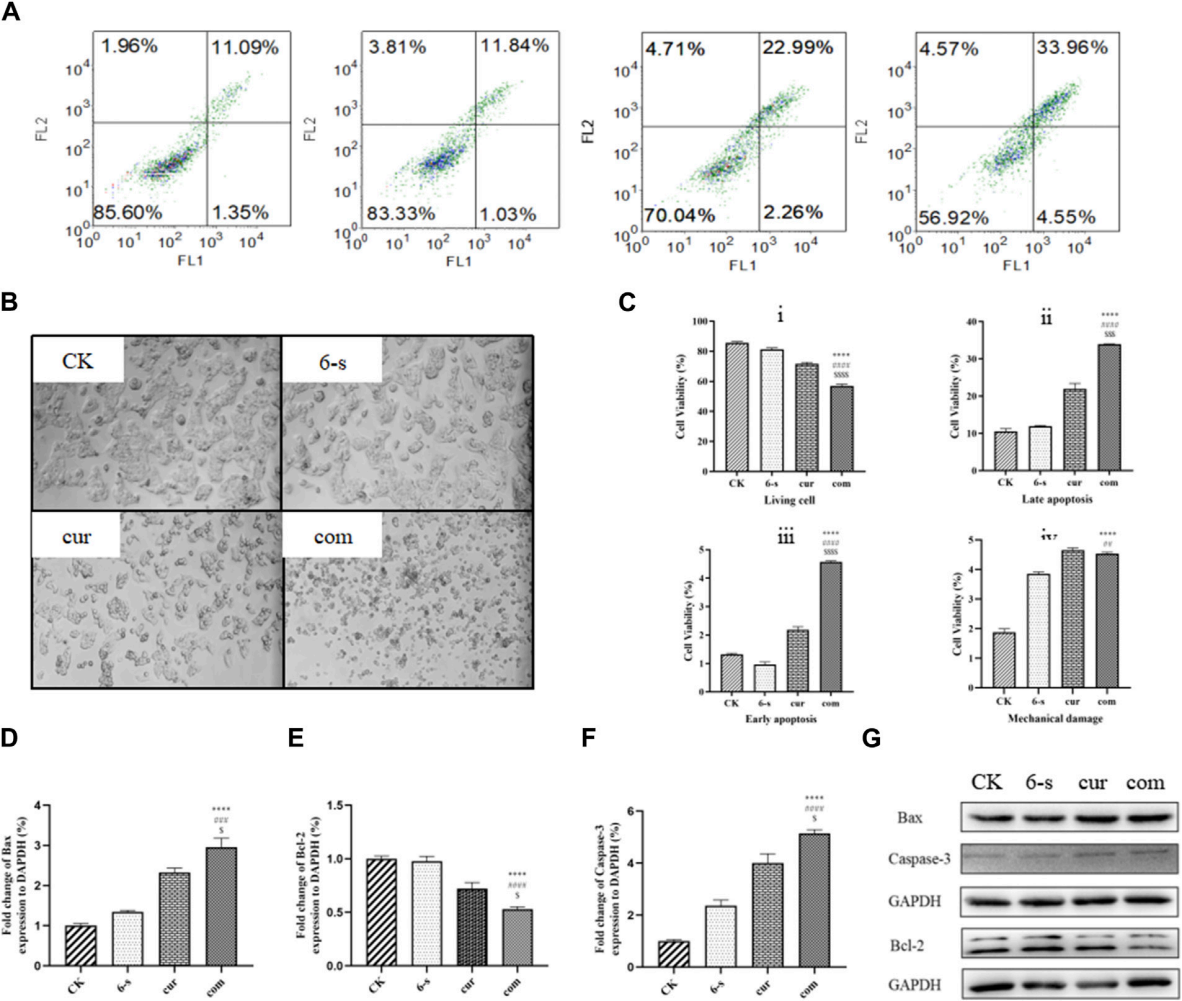
Combined treatment with 6-Shogaol and Curcumin selectively induces apoptosis in human hepatocellular carcinoma cells. **(A)** Hepatocellular carcinoma was treated with 5 μm 6-shogaol and 30 μm curcumin and apoptosis was observed. **(B)** The morphology of each group of cells was observed under light microscope. **(C)** Statistical analysis of living cell i), late apoptosis ii), early apoptosis iii) and mechanical damage iv) in each group of cells. Results were expressed as mean ± SD (n = 3). ^*^ *p* < 0.05, ^**^ *p* < 0.01, ^***^ *p* < 0.001, ^****^ *p* < 0.0001, calculated compared to control. ^#^ *p* < 0.05, ^##^ *p* < 0.01, ^###^ *p* < 0.001, ^####^ *p* < 0.0001 with respect to individual 6-shogaol dose and ^$^ *p* < 0.05, ^$$^ *p* < 0.01, ^$$$^ *p* < 0.001, ^$$$$^ *p* < 0.0001 with respect to individual curcumin dose. **(D–G)** Changes in the expression of apoptosis-related proteins Bax, Bcl-2 and Caspase-3 protein after combined treatment of cells with 6-Shogaol and Curcumin. Results were expressed as mean ± SD (n = 3). ^*^ *p* < 0.05, ^**^ *p* < 0.01, ^***^ *p* < 0.001, ^****^ *p* < 0.0001, calculated compared to control. ^#^ *p* < 0.05, ^##^ *p* < 0.01, ^###^ *p* < 0.001, ^####^ *p* < 0.0001 with respect to individual 6-shogaol dose and ^$^ *p* < 0.05, ^$$^ *p* < 0.01, ^$$$^ *p* < 0.001, ^$$$$^ *p* < 0.0001 with respect to individual curcumin dose.

Cell morphology was observed under light microscopy at ×100 magnification (Figure 4B). It was observed that the morphology of HepG2 cells in 6-shogaol and curcumin combination treatment group was unhealthy, compared to both the control and single drug groups. Cell shrinkage and fragmentation of apoptotic vesicles were observed in the cells of the combined treatment group, while the control group had normal cell morphology. In the control group, most of the HepG2 cells were either shuttle-shaped or irregular polygonal in shape and grew against the wall. In the low-dose group, cell spacing increased and a small number of cells became round; in the middle-dose group, intercellular gaps increased further and a large number of cells became edematous and rounded and smaller; while in the high-dose group, most of the cells were round and lost their original shape and the number of adherent cells decreased significantly with the disappearance of nuclei and the beginning of cytoplasmic diffusion.

#### 3.2.4 6-shogaol and curcumin combination to promote apoptosis in HepG2 cells

Cells were double-stained with membrane linked protein AnnexinV/PI after 24 h treatment and detected by flow cytometry (Figures 3A,C). As shown, the negative cells were 85.6, 83.3% and 70.1% in the control, 6-shogaol alone and curcumin alone groups, respectively, while 56.9% in the co-treated group. The apoptosis rate (early apoptosis, late apoptosis, and mechanical damage) was significantly higher in the combination-treated group, compared to the other groups and the cell mortality rate was lower in the control group.

#### 3.2.5 Combined use of 6-shogaol and curcumin to block the HepG2 cell cycle

**FIGURE 5:**
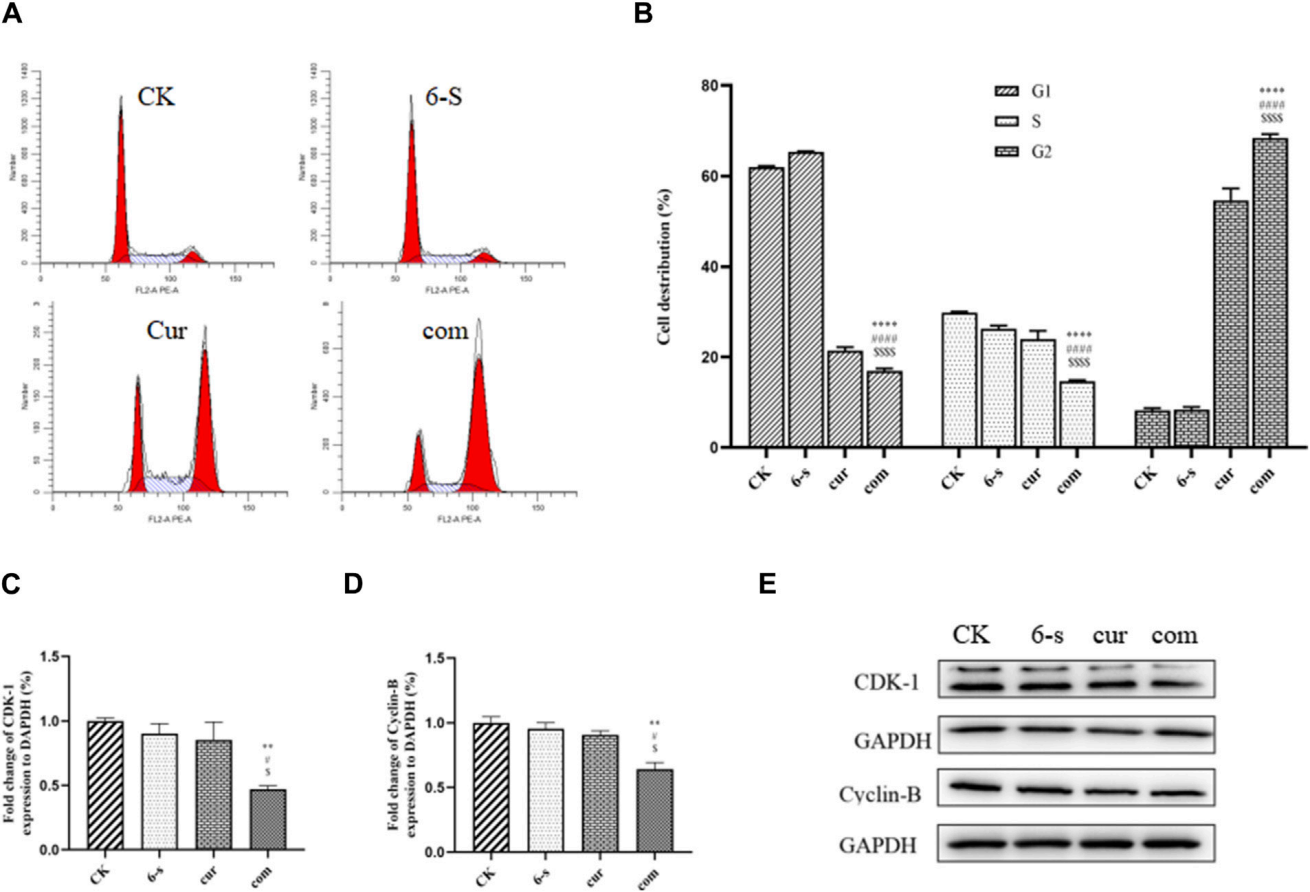
Combined treatment with 6-Shogaol and Curcumin induces cell cycle arrest in human hepatocellular carcinoma cells. **(A)** Cells were treated with 6-shogaol and curcumin alone and in combination to observe the cycle changes. **(B)** Statistical analysis of cell cycle changes in each group. The mean of the results is ±SD (n = 3). The synergistic group was compared with the other three groups, ^*^ *p* < 0.05,^**^ *p* < 0.01,^***^ *p* < 0.001, ^****^ *p* < 0.0001. **(C–E)** Changes in the expression of cycle-related proteins CDK-1 and Cyclin-B protein after combined treatment of cells with 6-Shogaol and Curcumin. Results were expressed as mean ± SD (n = 3). ^*^ *p* < 0.05, ^**^ *p* < 0.01, ^***^ *p* < 0.001, ^****^ *p* < 0.0001, calculated compared to control. ^#^ *p* < 0.05, ^##^ *p* < 0.01, ^###^ *p* < 0.001, ^####^ *p* < 0.001 with respect to individual 6-shogaol dose and ^$^ *p* < 0.05, ^$$^ *p* < 0.01, ^$$$^ *p* < 0.001, ^$$$$^ *p* < 0.0001 with respect to individual curcumin dose.

After 24 h of cell treatment, PI staining was performed and cell cycle changes were detected by flow cytometry (Figures 5A,B). As shown in the figure, 68.4% of G2 phase cells were found in the co-treatment group, compared to 8.2%, 8.4% and 54.1% in the control, 6-S and Cur groups, respectively. Compared with the other three groups, the G1 and S-phase cells were significantly lower and the G2 phase cells were significantly higher in the combined treatment group, indicating that the cells were blocked in the G2 phase.

#### 3.2.6 Effect of the combination of 6-shogaol and curcumin on HepG2 cell-associated proteins

**FIGURE 6:**
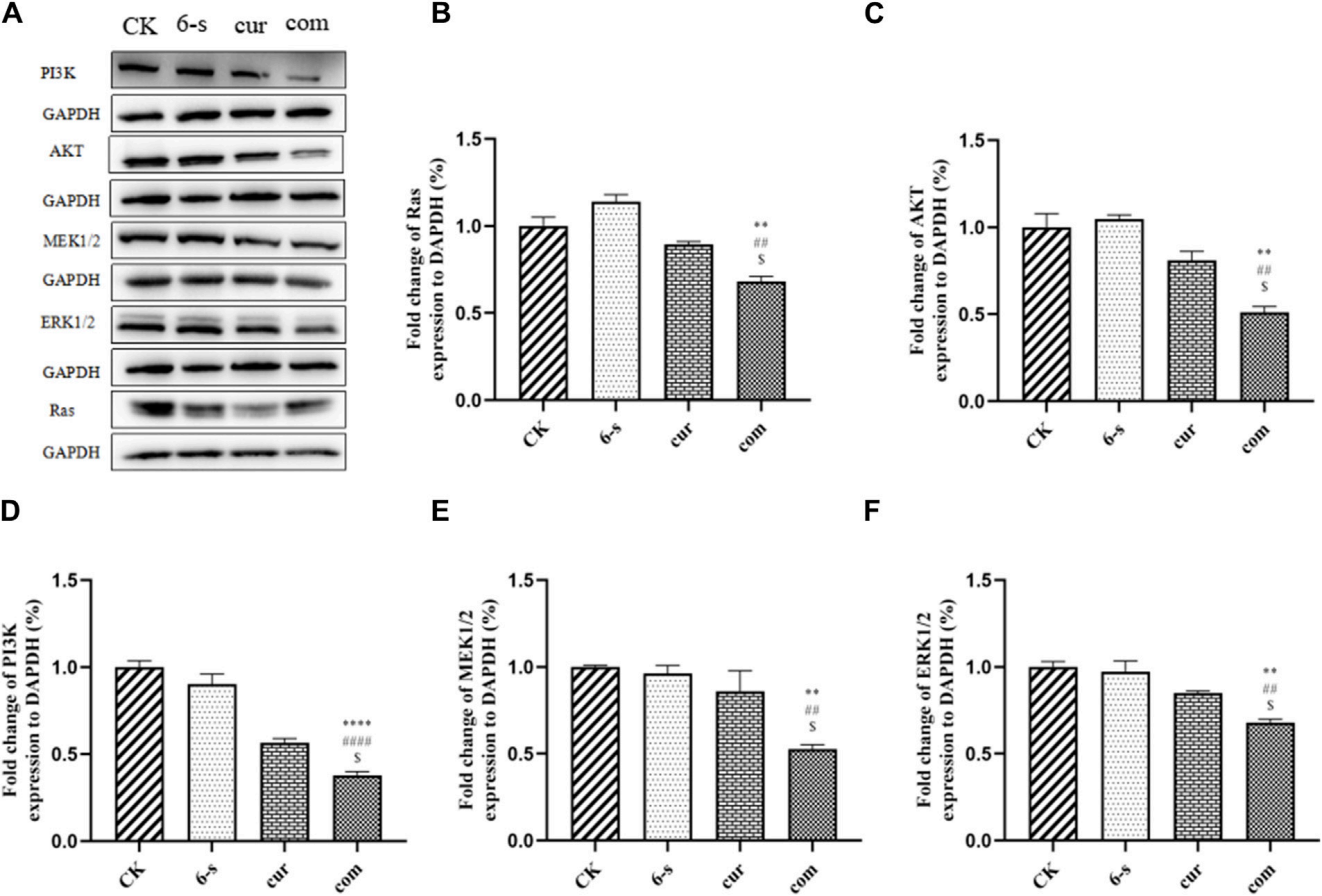
Effect of 6-Shogaol and curcumin on protein expression. **(A)** Expression of Ras, PI3K, AKT, MEK1/2 and ERK1/2 was analyzed by Western blot. **(B–F)** Quantitative analysis of protein expression in HepG2 cells. Results were expressed as mean ± SD (n = 3). ^*^ *p* < 0.05, ^**^ *p* < 0.01, ^***^ *p* < 0.001, ^****^ *p* < 0.0001 calculated compared to control. ^#^ *p* < 0.05, ^##^ *p* < 0.01, ^###^ *p* < 0.001, ^####^ *p* < 0.0001 with respect to individual 6-shogaol dose and ^$^ *p* < 0.05, ^$$^ *p* < 0.01, ^$$$^ *p* < 0.001, ^$$$$^ *p* < 0.0001 with respect to individual curcumin dose.

Western blotting assay was used to detect the effect of 6-shogaol and curcumin on the expression of related proteins. The results showed that both the individual and combined treatment groups affected protein expression to different degrees, with the combined treatment group having a more significant effect. Inhibition of protein expression in PI3/AKT signalling pathway and MAPK signalling pathway was observed (Figures 6A–F). In addition, pro-apoptotic protein Bax, apoptosis-related enzyme Caspase-3 and anti-apoptotic protein Bcl-2 were upregulated (Figures 4D–G) and cycle-related proteins Cyclin-B and CDK-1 were downregulated in the apoptosis-related proteins (Figures 5C–E). It is suggested that the combined action of 6-shogaol and curcumin against hepatocellular carcinoma may be related to the inhibition of PI3/AKT and MAPK signalling pathways, upregulation of Bax and Caspase-3 proteins, and downregulation of Bcl-2, Cyclin-B, and CDK-1 proteins.

## 4 Discussion

Characterised by insidious onset and lack of specific symptoms in the early stages, hepatocellular carcinoma is an extremely dangerous malignancy of the digestive system. From a molecular perspective, HCC is caused by the accumulation of multiple genomic and epigenomic alterations, which commonly include oncogenic mutations in the TERT promoter, AXIN1, TP53, and CTNNB1; amplification of chromosome 1q, 8q; and loss of 8p, 22p (Craig et al., 2020). These genetically altered genes are often associated with pathways such as Wnt-β-catenin, cell cycle regulation, AKT-mTOR, and MAPK (Lin et al., 2023). HepG2 cells are derived from human hepatocellular carcinoma cell lines and have the same biological activity as normal hepatocytes and remain stable during multiple passages. Its biochemical characteristics and biosynthetic capacity are similar to those of human normal hepatocytes and can be used to mimic human liver for functional and toxicological studies (Yang et al., 2019).

Web pharmacology is a discipline based on data analysis, database searching, and web construction. Network prediction models are constructed from published data. Topological analysis is used to predict drug targets and mechanisms to assess drug-organism interactions (Wang et al., 2021). Liu Shu et al. screened the two active ingredients of Tripterygium wilfordii against liver cancer, triptolide and celastrol, based on Traditional Chinese Medicine Systematic Pharmacology Database and Analysis Platform (TCMSP), and screened the differentially expressed genes through multiple disease databases to find the core substances and targets that mapped to each other, constructed the pharmacodynamic ingredient-target (C-T) network, the protein-protein interactions network (PPI), and performed GO and KEGG The C-T network and protein interaction network (PPI) were constructed, and GO and KEGG enrichment analyses were performed. It was found that the core targets such as PTGS2, CXCL8, TGFB1, and STAT3 were mainly enriched in the pathways related to cancer and several inflammatory factors, and that tretinoin and PTGS2 as well as tretinoin and PTGS2, CXCL8, and STAT3 had strong binding activities (Liu et al., 2021). Studying the interaction network between proteins can help to excavate the core regulatory genes, and there are already many protein interaction databases, and STRING database is one of them that covers the most species and has the largest interaction information. STRING database can retrieve a single protein by using its name, sequence and other formats. Searching for a single protein will give a network of all the proteins that interact with the protein. This function is more suitable for exploring the interaction of a protein, while inputting more than one protein at a time will only give the interaction network between the input proteins, which is more suitable for mining the interaction between the input proteins. For example, input all the differential genes identified by transcriptome data and analyze the interaction between these differential genes. After the search, we will get a network graph, which we call protein-protein interaction network, namely PPI network. DAVID is not only a biological database, but also an online analysis software. It can be used for differential analysis of genes and also for pathway enrichment. He can take the genes in the input list and associate them to biological annotations. In this study, 72 key targets for 6-shogaol and curcumin for the treatment of HCC were identified through the analysis of network topology and a search of databases. KEGG and GO enrichment analyses were performed on the key targets to explore the potential mechanism of combined action of 6-shogaol and curcumin to inhibit HCC. AKT1 (serine and threonine kinase AKT) is also called protein kinase B (PKB). AKT1 is activated by insulin and a variety of growth and survival factors and plays a key role in regulating cell growth and division and inhibiting apoptosis. There are three members in the AKT family, and AKT1 and AKT2 are widely distributed. As a key protein in PI3K/AKT/mTOR signaling pathway, AKT1 can prevent apoptosis and promote cell survival, which is considered to be a major factor in many types of cancer. Early findings of PI3K activity linked it to pathological cell growth and tumorigenesis, but it was not until 2004 that somatic mutations in PIK3CA were reported in cancer. Currently, genetic overactivation of PI3K/AKT signaling has been identified as one of the most common driving mechanisms in many cancers by high-throughput sequencing. Phosphatidylinositol 3-kinase (PI3Ks) protein family is involved in the regulation of cell proliferation, differentiation, apoptosis and glucose transport. Increased PI3K activity is often associated with a variety of cancers. PI3K phosphorylates phosphatidylinositol PI (a membrane phospholipid) at the third carbon atom of the inositol ring. PI accounted for a small proportion of cell membrane components, which was less than phosphatidylcholine, phosphatidylethanolamine and phosphatidylserine. But in the brain cell membrane, the content is more abundant, up to 10% of the total phospholipids. MAPK1 is a kinase that belongs to the MAPK1/2 family. This family consists of three main members: MAPK1 (ERK-2), MAPK2 (ERK-3), and MAPK3 (ERK-4). MAPK1 has various functions in cells, such as participating in the regulation of cell proliferation, differentiation, and apoptosis, the maintenance of cellular morphology, and the control of the cell cycle. In addition, MAPK1 has important biological significance, such as being closely related to the pathogenesis of tumorigenesis, neurodegenerative diseases and other diseases. MEK (Mitogen-activated protein kinase kinase, MAPKK) is a kinase enzyme which phosphorylates mitogen-activated protein kinases (MAPKs). The activated MAPK leads to the phosphorylation of downstream transcription factors that regulate various responses such as stress signaling, pathogen response, and hormone signaling. The enrichment analysis showed that 6-shogaol was mainly enriched in Ras, Fox0, Prolactin and C-type lectin receptor signalling pathways. The genetic alterations in cancer cells are tightly linked to signaling pathway dysregulation. Ras is a key molecule that control several tumorigenesis-related processes, and mutations in RAS genes often lead to unbiased intensification of signaling networks that fuel cancer progression. RAS exists as three major isoforms, Kirsten rat sarcoma (KRAS), Harvey rat sarcoma (HRAS) and neuroblastoma rat sarcoma (NRAS). Mutations of these genes account for approximately 30% of all cancers. Among them, KRAS mutations are the most common, responsible for 85%, followed by NRAS (12%) and HRAS (3%) (Martin et al., 2013; Valtorta et al., 2013; Temraz et al., 2015; Mármol et al., 2017). The Ras signalling pathway is closely related to tumorigenesis and growth; drug design for important targets of this signalling pathway is a hot topic in current anti-tumor drug research. Mutant Ras proteins regulate several downstream effectors; many of which are aberrantly activated during cancer progression and the main signalling pathways mediated by them are the RAF/MEK/ERK kinase pathway and the PI3K/AKT signalling pathway. These two pathways, respectively regulate cell survival and cell proliferation (Takacs et al., 2020). Other Ras-driven signalling pathways: the TIAM1/RAC/PAK pathway mainly controls cytoskeletal rearrangements in certain cells; the RalGDS/Ral pathway mainly affects membrane transport; the NORE1/RASSF1/MST signalling pathway is a regulator of the cell death process; and PLC/PKC molecule-mediated signalling pathway affects calcium-dependent signalling in cancer cells (Kadamur and Ross, 2013; Donninger et al., 2016; Semenova et al., 2017; Yoshizawa et al., 2017). PI3K proteins belong to the lipid kinase family and can be divided into class I, II and III isoforms, among which, type I plays a very important role in tumours (Jiang et al., 2020). Akt is a serine threonine kinase downstream of PI3K and belongs to the AGC subfamily of protein kinases (Shi et al., 2019). In the classical PI3K/Akt activation model, activated phosphorylated Akt phosphorylates several downstream substrates, including the FOX transcription factor O subfamily (FOXO), mammalian target of rapamycin protein (mTOR), glycogen synthase kinase 3β (GSK3β), and others, regulating various key cellular activities, such as cell growth, proliferation, survival, genome stabilization, and glucose metabolism. The PI3K/Akt signalling pathway is aberrantly activated in approximately 50% of HCC and plays a critical role in the development of hepatocellular carcinoma (Rahmani et al., 2019). One explanation for the oncogenicity of mutant Ras proteins in a constitutively active GTP-bound state is the initiation of an increased MAPK signalling pathway (Guo et al., 2020). Extracellular signal-regulated kinase (ERK), a signal transduction protein of the MAPK family that includes two isoforms (ERK1 and ERK2) is highly expressed in HCC and regulates the development of HCC (Saidak et al., 2017).

As the results of network pharmacology are themselves a prediction only, such results cannot be applied without experimental validation. To further elucidate the mechanism of action of 6-shogaol and curcumin in ameliorating hepatocellular carcinoma, we conducted *in vitro* experiments. Inhibition of cancer cell growth and proliferation is the key to the prevention and treatment of cancer and the Chou-Talalay method is a scientific method for evaluating the combination of drugs. The results showed that 6-shogaol and curcumin alone and in combination can inhibit the growth and proliferation of HepG2 cells while the combination of different components can inhibit various cancer cell lines; for example, microcystin-LR, chylindrospermopsin and their combinations can affect the cytotoxicity and morphology of the human hepatocyte line HepG2 (Gutierrez-Praena et al., 2019). The combination of curcumin and rhodopsin synergistically inhibits breast cancer cell proliferation and invasion through upregulation of miR-34a (Guo et al., 2013). In this study, 6-shogaol and curcumin showed an effective combination that exerted synergistic inhibition in HepG2 cancer cell lines, thus proving effective against hepatocellular carcinoma tumour entities *in vitro*. The cell cycle is a series of events that occur in a single cell that drive cell division and give rise to 2 cells. The cell cycle has important implications for tumour cell proliferation, metastasis, and recurrence (Yu and Zuo, 2013). In current cancer therapy, cell cycle regulation is mainly concerned with controlling the expression of relevant genes and the activity of intracellular enzymes, proteins or signalling factors (Miyazono et al., 2010). In this study, we measured the effects of 6-shogaol and curcumin, alone and in combination, on the cell phase of the hepatocellular carcinoma cell line (HepG2) using flow cytometry. The results of this study showed that 6-shogaol and curcumin alone and in combination blocked the cell cycle in the G1 and S phases. Therefore, 6-shogaol and curcumin, alone and in combination, can inhibit the proliferation of hepatocellular carcinoma *in vitro*. The binding of cell cycle proteins and cell cycle protein-dependent kinase (CDK) is closely associated with cell cycle transition (Palmer et al., 2019). The transition from G1 to S phase results in a cascade of cell cycle protein D, CDK-4 complexes binding to CDK-6, and cell cycle protein B, while CDK-1 complexes shift from G2 to M during mitosis (Hu et al., 2019). When DNA is damaged, the G2 phase detection site causes the cell to enter mitosis for auto-repair. When cyclin-B and cyclin-A form a complex with CDK-1, it is extremely important to produce a cascade activation of the M-phase transition of the pair. In our experiments, the combination group was found to significantly downregulate the expression of cyclin-B protein and CDK-1. This suggests that DIOS may induce G2/M phase arrest through downregulation of cyclin-B/CDK-1. Ma and coworkers. found that DIOS can block the HepG2 cell cycle in G2/M phase through downregulation of cyclin-B/CDK-1 expression (Ma and Zhang, 2020). The role of apoptosis in cancer has received much attention (Yang et al., 2013) and the resistance to apoptosis is widely regarded as an acquired characteristic of cancer cells, conferring them a survival advantage that promotes tumour evolution and growth, as well as leading to therapeutic failure of tumours (Lewis and Mohanty, 2010). Therefore, in this study, we determined the apoptosis of the hepatocellular carcinoma cell line (HepG2 cells) by using flow cytometry with 6-shogaol and curcumin alone and in combination and found that the combination group induced apoptosis in Hep G2 cells, a result that suggested that the combination of 6-shogaol and curcumin inhibited the development of HCC. Apoptosis-related proteins include anti-apoptotic proteins (Bcl-2), pro-apoptotic proteins (Bax), and apoptotic effectors (For example, caspase-9 and caspase-3). These factors play an important role in apoptosis. The molecular mechanisms of apoptosis in hepatocellular carcinoma are very complex and the main apoptotic pathways are the endogenous apoptotic pathway, the exogenous apoptotic pathway, and the common apoptotic pathway (Dimri and Satyanarayana, 2021). The Bcl-2 protein family is the main regulator of endogenous apoptosis and is usually divided into two major groups. The first group is the anti-apoptotic proteins (For example, Bcl-2, Bcl-XL, MCL1, etc.), which all contain four different BH homologous domains and can exert anti-apoptotic effects by antagonizing pro-apoptotic proteins. The second group of pro-apoptotic proteins includes both Bax and Bak, which also contain four BH homologous structural domains, and BH3-only proteins (For example, t BID, BAD, and PUMA), which contain only one BH3 structural domain (Galluzzi et al., 2018). The results of this experiment showed that 6-shogaol and curcumin, alone as well as in combination, was able to promote apoptosis, inhibiting the survival of hepatocellular carcinoma. The morphological observations in this study were similar to the changes reported by other authors (Jayant et al., 2017), with cell shrinkage, fragmentation of apoptotic vesicles and cytoplasmic spreading in the 6-shogaol and curcumin alone as well as in the combined group. It was noted that 6-shogaol and curcumin, alone as well as in combination, caused cell death through autophagy and apoptosis.

The mechanism by which 6-shogaol and curcumin combine to induce cancer cell death is still in the beginning stages. Interestingly, we confirmed by Western Blot test that both 6-shogaol and curcumin exert anti-tumour effects through inhibition of the Ras-mediated PI3K/AKT and MAPK signalling pathways, which partly explained the synergistic effects observed here. We found that this combination induced cell cycle and apoptosis in hepatocellular carcinoma. Shen and coworkers assessed the activity of key effectors in the pathway and found that both the Ras/MAPK and phosphatidylinositol 4,5-bisphosphate 3-kinase (PI3K)/Akt signalling pathways were regulated by HRAS activating mutations (Shen et al., 2016). Our data suggested that this mechanism might also apply to the combination of 6-shogaol and curcumin.

## 5 Conclusion

We constructed a network of interactions between 6-shogaol and curcumin, and hepatocellular carcinoma to predict their pathways of action and network pharmacological functions. Seventy-two targets were found to be closely associated with the effects of 6-shogaol and curcumin in the treatment of HCC. Detailed studies on HepG2 cell models showed that both 6-shogaol and curcumin inhibited the proliferative survival of the cells and that the combined effect showed a stronger inhibition than the action alone. Morphological studies on the cells supported the cytotoxic results obtained. In addition, 6-shogaol and curcumin induced cell cycle arrest in the G2/M phase by down-regulating Cyclin-B, CDK-1, Bcl-2 and up-regulating Bax, and promoted apoptosis. Further investigation of signalling pathways showed that 6-shogaol and curcumin acted synergistically to inhibit Ras protein expression and regulate PI3K/AKT and MAPK signalling pathways. These results suggest that 6-shogaol in combination with curcumin is a potential combination therapy for HCC.

## Data Availability

The datasets presented in this study can be found in online repositories. The names of the repository/repositories and accession number(s) can be found in the article/supplementary material.

## Abbreviations

BP, biological process, CC, cellular component, CI, cardiac index, DRI, dosereductionindex, FBS, fetal bovine serum, HCC, hepatocellular carcinoma, HPLC, high-performance liquid chromatography, KEGG, Kyoto Encyclopedia of Genes and Genomes; MAPK, mitogen-activated protein kinase; MEM, minimum Eagle’s medium; MF, molecular function; MTT, 3-(4,5-dimethyl-2-thiazolyl)-2,5-diphenyl-2-H-tetrazolium bromide; PI3K/AKT, Phosphatidylinositide 3-kinases/Protein Kinase B; GO, Gene Ontology.

## References

[B1] Bai C. H. Zhao J. Q. Su J. L. Chen J. X. Cui X. M. Sun M. Q. (2022). Curcumin induces mitochondrial apoptosis in human hepatoma cells through BCLAF1-mediated modulation of PI3K/AKT/GSK-3β signaling. Life Sci. 306, 120804. 10.1016/j.lfs.2022.120804 35882275

[B2] Bawadood A. S. Al-Abbasi F. A. Anwar F. El-Halawany A. M. Al-Abd A. M. (2020). 6-shogaol suppresses the growth of breast cancer cells by inducing apoptosis and suppressing autophagy via targeting notch signaling pathway. Biomed. Pharmacother. 128, 110302. 10.1016/j.biopha.2020.110302 32505819

[B3] Couri T. Pillai A. (2019). Goals and targets for personalized therapy for HCC. Hepatol. Int. 13 (2), 125–137. 10.1007/s12072-018-9919-1 30600478

[B4] Craig A. J. Von Felden J. Garcia-Lezana T. Sarcognato S. Villanueva A. (2020). Tumour evolution in hepatocellular carcinoma. Nat. Rev. Gastroenterol. Hepatol. 17 (3), 139–152. 10.1038/s41575-019-0229-4 31792430

[B5] Dalsasso R. R. Valencia G. A. Monteiro A. R. (2022). Impact of drying and extractions processes on the recovery of gingerols and shogaols,the main bioactive compounds of ginger. Food Res. Int. 154, 111043. 10.1016/j.foodres.2022.111043 35337584

[B6] Dewangan J. Tandon D. Srivastava S. Verma A. K. Yapuri A. Rath S. K. (2017). Novel combination of salinomycin and resveratrol synergistically enhances the anti-proliferative and pro-apoptotic effects on human breast cancer cells. Apoptosis 22 (10), 1246–1259. 10.1007/s10495-017-1394-y 28748373

[B7] Dimri M. Satyanarayana A. (2021). Molecular signaling pathways and therapeutic targets in hepatocellular carcinoma. Adv. Cancer Res. 149, 491–101. 10.3390/cancers12020491 PMC7072513 32093152

[B8] Donninger H. Schmidt M. L. Mezzanotte J. Barnoud T. Clark G. J. (2016). Ras signaling through RASSF proteins. Semin. Cell Dev. Biol. 58, 86–95. 10.1016/j.semcdb.2016.06.007 27288568 PMC5034565

[B9] Galluzzi L. Vitale I. Aaronson S. A. Abrams J. M. Kroemer G. Agostinis P. (2018). Molecular mechanisms of cell death: recommendations of the nomenclature committee on cell death 2018. Cell Death Differ. 25 (3), 486–541. 10.1038/s41418-017-0012-4 29362479 PMC5864239

[B10] Guo J. L. Li W. P. Shi H. L. Xie X. H. Li L. S. Tang H. L. (2013). Synergistic effects of curcumin with emodin against the proliferation and invasion of breast cancer cells through upregulation of miR-34a. Mol. Cell Biochem. 382 (1-2), 103–111. 10.1007/s11010-013-1723-6 23771315

[B11] Guo Y. J. Pan W. W. Liu S. B. Shen Z. F. Xu Y. Hu L. L. (2020). ERK/MAPK signalling pathway and tumorigenesis. Exp. Ther. Med. 19 (3), 1997–2007. 10.3892/etm.2020.8454 32104259 PMC7027163

[B12] Gutierrez-Praena D. Guzman-Guillen R. Pichardo S. Moreno F. J. Vasconcelos V. Jos A. (2019). Cytotoxic and morphological effects of microcystin-LR, cylindrospermopsin, and their combinations on the human hepatic cell line HepG2. Environ. Toxicol. 34 (3), 240–251. 10.1002/tox.22679 30461177

[B13] Hsu Y. L. Hung J. Y. Tsai Y. M. Tsai E. M. Huang M. S. Hou M. F. (2015). 6-Shogaol, an active constituent of dietary ginger, impairs cancer development and lung metastasis by inhibiting the secretion of CC-chemokine ligand 2 (CCL2) in tumor-associated dendritic cells. J. Agric. Food Chem. 63 (6), 1730–1738. 10.1021/jf504934m 25621970

[B14] Hu R. F. Sun X. B. (2019). Design of new traditional Chinese medicine herbal formulae for treatment of type 2 diabetes mellitus based on network pharmacology. Chin. J. Nat. Med. 15 (6), 436–441. 10.1016/S1875-5364(17)30065-1 28629533

[B15] Hu X. Eastman A. E. Guo S. Q. (2019). Cell cycle dynamics in the reprogramming of cellular identity. FEBS Lett. 593 (20), 2840–2852. 10.1002/1873-3468.13625 31562821

[B16] Ikeda M. Morizane C. Ueno M. Okusaka T. Ishii H. Furuse J. (2018). Chemotherapy for hepatocellular carcinoma: current status and future perspectives. Jpn. J. Clin. Oncol. 48 (2), 103–114. 10.1093/jjco/hyx180 29253194

[B17] Ivane N. M. A. Elyse F. K. R. Haruna S. A. Pride N. Richard E. Foncha A. C. (2022). The anti-oxidative potential of ginger extract and its constituent on meat protein isolate under induced Fenton oxidation. J. Proteomics 269, 104723. 10.1016/j.jprot.2022.104723 36096434

[B18] Jayant D. Divya T. Sonal S. Ajeet K. Ashok Y. Srikanta K. (2017). Novel combination of salinomycin and resveratrol synergistically enhances the anti-proliferative and pro-apoptotic effects on human breast cancer cells. Apoptosis 22, 1246–1259. 10.1007/s10495-017-1394-y 28748373

[B19] Jiang N. N. Dai Q. J. Su X. R. Fu J. J. Feng X. C. Peng J. (2020). Role of PI3K/AKT pathway in cancer: the framework of malignant behavior. Mol. Biol. Rep. 47 (6), 4587–4629. 10.1007/s11033-020-05435-1 32333246 PMC7295848

[B20] Jiao W. Y. Mi S. Sang Y. X. Jin Q. X. Chitrakar B. Wang X. H. (2021). Integrated network pharmacology and cellular assay for the investigation of an anti-obesity effect of 6-shogaol. Food Chem. 374, 131755. 10.1016/j.foodchem.2021.131755 34883426

[B21] Kadamur G. Ross E. M. (2013). Mammalian phospholipase C. Annu. Rev. Physiol. 75, 127–154. 10.1146/annurev-physiol-030212-183750 23140367

[B22] Kifer D. Jaksic D. Klaric M. S. (2020). Assessing the effect of mycotoxin combinations: which mathematical model is (the most) appropriate? Toxins (Basel). 12 (3), 153. 10.3390/toxins12030153 32121330 PMC7150917

[B23] Kumar N. Afjei R. Massoud T. F. Paulmurugan R. (2018). Comparison of cell-based assays to quantify treatment effects of anticancer drugs identifies a new application for Bodipy-L-cystine to measure apoptosis. Sci. Rep. 8, 16363. 10.1038/s41598-018-34696-x 30397244 PMC6218539

[B24] Lewis J. R. Mohanty S. R. (2010). Nonalcoholic fatty liver disease: a review and update. Dig. Dis. Sci. 55 (3), 560–578. 10.1007/s10620-009-1081-0 20101463

[B25] Li W. Sun L. K. Lei J. J. Wu Z. Ma Q. Y. Wang Z. (2020). Curcumin inhibits pancreatic cancer cell invasion and EMT by interfering with tumor-stromal crosstalk under hypoxic conditions via the IL-6/ERK/NF-kappa B axis. Oncol. Rep. 44 (1), 382–392. 10.3892/OR.2020.7600 32377752

[B26] Lin Z. Z. Hu M. Hsu C. Wu Y. M. Lu Y. S. Ho J. A. (2023). Synergistic efficacy of telomerase-specific oncolytic adenoviral therapy and histone deacetylase inhibition in human hepatocellular carcinoma. Cancer Lett. 556, 216063. 10.1016/j.canlet.2023.216063 36669725

[B27] Liu J. Fan J. Y. Zhou F. L. Xiong Y. Shi H. J. Wang X. W. (2022). Berberine combined with formononetin inhibits migration of nasopharyngeal carcinoma cells through the MAPK/ERK1/2 signaling pathway. J. Funct. Foods 93, 105088. 10.1016/j.jff.2022.105088

[B28] Liu S. Lu J. D. Lv Y. T. Yu C. Y. Xiong G. L. (2021). Study of the molecular mechanism of Tripterygium wilfordii inhibition of liver cancer based on network pharmacology and its preliminary verification. J. B Univ. Technol. 48 (5), 66–75. 10.13543/j.bhxbzr.2021.05.009

[B29] Liu Y. Liu J. C. Zhang Y. Q. (2019). Research progress on chemical constituents of Zingiber officinale Roscoe. Biomed. Res. Int. 2019, 5370823. 10.1155/2019/5370823 31930125 PMC6942719

[B30] Ma A. Q. Zhang R. (2020). Diosmetin inhibits cell proliferation, induces cell apoptosis and cell cycle arrest in liver cancer. Cancer Manag. Res. 12, 3537–3546. 10.2147/CMAR.S240064 32547191 PMC7244522

[B31] Mármol I. Sánchez-de-Diego C. Pradilla D. A. Cerrada E. Rodriguez Y. M. J. (2017). Colorectal carcinoma: a general overview and future perspectives in colorectal cancer. Int. J. Mol. Sci. 18 (1), 197. 10.3390/ijms18010197 28106826 PMC5297828

[B32] Martin T. D. Chen X. W. Kaplan R. E. Saltiel A. R. Walker C. L. Reiner D. J. (2013). Ral and Rheb GTPase activating proteins integrate mTOR and GTPase signaling in aging, autophagy, and tumor cell invasion. Mol. Cell 53 (2), 209–220. 10.1016/j.molcel.2013.12.004 PMC3955741 24389102

[B33] Miyazono K. Kamiya Y. Morikawa M. (2010). Bone morphogenetic protein receptors and signal transduction. J. Biochem. 147, 35–51. 10.1093/jb/mvp148 19762341

[B34] Nazim U. M. Park S. Y. (2019). Attenuation of autophagy flux by 6-Shogaol sensitizes human liver cancer cells to TRAIL-induced apoptosis via p53 and ROS. Int. J. Mol. Med. 43 (2), 701–708. 10.3892/ijmm.2018.3994 30483736 PMC6317668

[B35] Palmer N. Talib S. Z. A. Kaldis P. (2019). Diverse roles for CDK-associated activity during spermatogenesis. FEBS Lett. 593 (20), 2925–2949. 10.1002/1873-3468.13627 31566717 PMC6900092

[B36] Rahmani F. Ziaeemehr A. Shahidsales S. Gharib M. Khazaei M. Ferns G. A. (2019). Role of regulatory miRNAs of the PI3K/Akt/mTOR signaling in the pathogenesis of hepatocellular carcinoma. J. Cell Physiol. 235 (5), 4146–4152. 10.1002/jcp.29333 31663122

[B37] Saidak Z. Giacobbi A. S. Louandre C. Sauzay C. Mammeri Y. Galmiche A. (2017). Mathematical modelling unveils the essential role of cellular phosphatases in the inhibition of RAF-MEK-ERK signalling by sorafenib in hepatocellular carcinoma cells. Cancer Lett. 392, 1–8. 10.1016/j.canlet.2017.01.038 28161506

[B38] Sani I. K. Marashi S. H. Kalalinia F. (2015). Solamargine inhibits migration and invasion of human hepatocellular carcinoma cells through down-regulation of matrix metalloproteinases 2 and 9 expression and activity. Toxicol Vitro 29 (5), 893–900. 10.1016/j.tiv.2015.03.012 25819016

[B39] Semenova G. Stepanova D. S. Dubyk C. Handorf E. Deyev S. M. Lazar A. J. (2017). Targeting group I p21-activated kinases to control malignant peripheral nerve sheath tumor growth and metastasis. Oncogene 36 (38), 5421–5431. 10.1038/onc.2017.143 28534510 PMC5608634

[B40] Shen J. Tsoi H. Liang Q. Chu E. S. H. Liu D. Yu A. C. S. (2016). Oncogenic mutations and dysregulated pathways in obesity-associated hepatocellular carcinoma. Oncogene 35 (49), 6271–6280. 10.1038/onc.2016.162 27132506 PMC5153568

[B41] Shi X. Wang J. J. Lei Y. Cong C. F. Tan D. L. Zhou X. R. (2019). Research progress on the PI3K/Akt signaling pathway in gynecological cancer (Review). Mol. Med. Rep. 19 (6), 4529–4535. 10.3892/mmr.2019.10121 30942405 PMC6522820

[B42] Specian A. F. L. erpeloni J. M. Tuttis K. Ribeiro D. L. iliao H. L. Varanda E. A. (2016). LDH, proliferation curves and cell cycle analysis are the most suitable assays to identify and characterize new phytotherapeutic compounds. Cytotechnology 68 (6), 2729–2744. 10.1007/s10616-016-9998-6 27344148 PMC5101344

[B43] Takacs T. Kudlik G. Kurilla A. Szeder B. Buday L. Vas V. (2020). The effects of mutant Ras proteins on the cell signalome. Cancer Metastasis Rev. 39 (4), 1051–1065. 10.1007/s10555-020-09912-8 32648136 PMC7680337

[B44] Tang Y. L. Sun L. Y. Wei J. C. Sun C. Gan C. Y. Xie X. F. (2022). Network pharmacology identification and *in vivo* validation of key pharmacological pathways of Phyllanthus reticulatus (Euphorbiaceae) leaf extract in liver cancer treatment. J. Ethnopharmacol. 297, 1115479. 10.2139/ssrn.4076529 35777610

[B45] Temraz S. Mukherji D. Shamseddine A. (2015). Dual inhibition of MEK and PI3K pathway in KRAS and BRAF mutated colorectal cancers. Int. J. Mol. Sci. 16, 22976–22988. 10.3390/ijms160922976 26404261 PMC4613347

[B46] Valtorta E. Misale S. Bianchi S. M. Nagtegaal I. D. Paraf F. Lauricella F. (2013). KRAS gene amplification in colorectal cancer and impact on response to EGFR-targeted therapy. Int. J. Cancer 133, 1259–1265. 10.1002/ijc.28106 23404247

[B47] Wang X. Wang N. Cheng F. Lao L. Li C. Feng Y. (2015). Chinese medicines for prevention and treatment of human hepatocellular carcinoma: current progress on pharmacological actions and mechanisms. J. Integr. Med. 13 (3), 142–164. 10.1016/S2095-4964(15)60171-6 26006028

[B48] Wang X. Wang Z. Y. Zheng J. H. Li S. (2021). TCM network pharmacology: a new trend towards combining computational, experimental and clinical approaches. Chin. J. Nat. Med. 19 (1), 1–11. 10.1016/S1875-5364(21)60001-8 33516447

[B49] Yang J. Fan J. Li Y. Li F. H. Chen P. K. Fan Y. B. (2013). Genome-wide RNAi screening identifies genes inhibiting the migration of glioblastoma cells. PLoS One 8 (4), e61915. 10.1371/journal.pone.0061915 23593504 PMC3625150

[B50] Yang Z. C. Huang W. Zhang J. S. Xie M. Wang X. W. (2019). Baicalein improves glucose metabolism in insulin resistant HepG2 cells. Eur. J. Pharmacol. 854, 187–193. 10.1016/j.ejphar.2019.04.005 30970232

[B51] Yoshizawa R. Umeki N. Yanagawa M. Murata M. Sako Y. (2017). Single-molecule fluorescence imaging of RalGDS on cell surfaces during signal transduction from Ras to Ral. Biophys. Physicobiol 14, 75–84. 10.2142/biophysico.14.0_75 28744424 PMC5515350

[B52] Yu X. K. Zuo Q. (2013). Micro RNAs in the regeneration of skeletal muscle. Front. Biosci. Landmark Ed. 18, 608–615. 10.2741/4124 23276946

[B53] Zhang J. L. Wang X. M. Yang J. F. Guo L. N. Wang X. L. Song B. (2020). Novel diosgenin derivatives containing 1,3,4-oxadiazole/thiadiazole moieties as potential antitumor agents: design, synthesis and cytotoxic evaluation. Eur. J. Med. Chem. 186, 111897. 10.1016/j.ejmech.2019.111897 31761382

[B54] Zhao Z. W. Su J. L. Zhao J. Q. Chen J. X. Cui X. M. Sun M. Q. (2022). Curcumin inhibits invasion and metastasis of human hepatoma cells through Bclaf1-mediated Wnt/β-catenin signalling. Food Agr. Immunol. 33 (1), 664–676. 10.1080/09540105.2022.2113864

[B55] Zheng R. S. Zhang S. W. Zeng H. M. Wang S. M. Sun K. X. Chen R. (2022). Cancer incidence and mortality in China, 2016. J. Natl. Cancer Cent. 2 (1), 1–9. 10.1016/j.jncc.2022.02.002 39035212 PMC11256658

[B56] Zhou X. Afzal S. Wohlmuth H. Munch G. Leach D. Low M. (2022). Synergistic anti-inflammatory activity of ginger and turmeric extracts in inhibiting lipopolysaccharide and interferon-gamma-induced proinflammatory mediators. Molecules 27 (12), 3877. 10.3390/molecules27123877 35745000 PMC9229778

[B57] Zhou X. Seto S. W. Chang D. Kiat H. Razmovski-Naumovski V. Chan K. (2016). Synergistic effects of Chinese herbal medicine: a comprehensive review of methodology and current research. Front. Pharmacol. 7, 201. 10.3389/fphar.2016.00201 27462269 PMC4940614

[B58] Zhu G. L. Liu X. L. Li H. J. Yan Y. Hong X. P. Lin Z. D. (2018). Kaempferol inhibits proliferation, migration, and invasion of liver cancer HepG2 cells by down-regulation of microRNA-21. Int. J. Immunopathol. Pharmacol. 32, 2058738418814341. 10.1177/2058738418814341 30477356 PMC6259061

